# Letter to the editor: enhancing prehabilitation protocols in frail older adults undergoing joint replacement - methodological insights from a pilot randomized controlled trial

**DOI:** 10.1016/j.tjfa.2025.100065

**Published:** 2025-07-11

**Authors:** Xinrui Sun, Fei Gao

**Affiliations:** a Department of Basic Medical Sciences, Fujian Medical University, Fuzhou, China; b Department of Anesthesiology, Shengli Clinical Medical College of Fujian Medical University; Department of Anesthesiology, Fuzhou University Affiliated Provincial Hospital, Fuzhou, China

Dear editor,

We read with great interest the innovative study "Getting fit for hip and knee replacement: The Fit-Joints multimodal intervention for frail patients with osteoarthritis – a pilot randomized controlled trial".While applauding the scientific rigour of this preoperative rehabilitation protocol for frail arthroplasty patients, we would like to propose several methodological considerations to enhance future research in this critical area [1].

Firstly, the study did not detail the pain levels of the participants. Given that chronic pain directly influences multiple frailty domains through mechanisms including sleep disruption, nutritional compromise [2], and mental health deterioration [3], standardized pain documentation would strengthen the interpretation of frailty metrics. We recommend systematic recording of analgesic regimens (including dosage adjustments and medication classes) alongside validated pain assessment tools. This would facilitate analysis of potential confounding effects from pharmacodynamic variability in analgesics [4], Particularly given this cohort's advanced mean age of 74 years - where prolonged observation periods may potentiate anxiety-related psychological comorbidities that accelerate frailty progression.

We also noted that the time points used in the study were one week preoperatively, 6 weeks and 6 months postoperatively, and the 6-week postoperative assessment timeline warrants scrutiny. We reviewed the literature and found that residual anaesthetic effects on neurological function - including postoperative cognitive dysfunction and sensorimotor impairment - may persist beyond this time frame [5], even some effects may persist for weeks or even months. We, therefore, recommend incorporating neurological and functional assessments (e.g.Montreal Cognitive Assessment, timed up-and-go test) at subsequent time points to differentiate between true frailty progression and transient perioperative neurophysiological changes. This distinction holds particular clinical relevance given the established correlation between persistent cognitive impairment and long-term functional decline in elderly surgical patients.

Last but not least, we wish to highlight the potential confounding effects of multimorbidity on frailty trajectories. While cardiovascular disease and diabetes mellitus are recognized accelerators of frailty phenotypes [6], their specific interactions with postoperative recovery remain under-characterized in orthopaedic populations. Stratification by comorbidity burden (e.g., using Charlson Comorbidity Index scores) could yield valuable insights into differential treatment responses and inform personalized rehabilitation protocols.

Although there are some minor flaws, it cannot be denied that the study makes a substantial contribution to perioperative care paradigms for frail older individuals. Moreover, our suggestions make an already perfect article look even better. Hopefully, in the future, we will see more medical professionals, government officials, and social workers working together to make healthcare optimize healthcare delivery for people all over the world.

## Funding information

No funding was received for conducting this study.

## CRediT authorship contribution statement

**Xinrui Sun:** Writing – review & editing, Writing – original draft. **Fei Gao:** Writing – review & editing.

## Declaration of competing interest

The authors declare the following financial interests/personal relationships which may be considered as potential competing interests:

Fei Gao reports article publishing charges was provided by Fujian Provincial Hospital. Fei Gao reports a relationship with Fujian Provincial Hospital that includes: employment. The authors declare that they have no competing interests. If there are other authors, they declare that they have no known competing financial interests or personal relationships that could have appeared to influence the work reported in this paper.
